# Huntington's Disease and its therapeutic target genes: a global functional profile based on the HD Research Crossroads database

**DOI:** 10.1186/1471-2377-12-47

**Published:** 2012-06-28

**Authors:** Ravi Kiran Reddy Kalathur, Miguel A Hernández-Prieto, Matthias E Futschik

**Affiliations:** 1 Centro de Biomedicina Molecular e Estrutural, Campus de Gambelas, Universidade do Algarve, Faro, Portugal

## Abstract

**Background:**

Huntington’s disease (HD) is a fatal progressive neurodegenerative disorder caused by the expansion of the polyglutamine repeat region in the *huntingtin* gene. Although the disease is triggered by the mutation of a single gene, intensive research has linked numerous other genes to its pathogenesis. To obtain a systematic overview of these genes, which may serve as therapeutic targets, CHDI Foundation has recently established the HD Research Crossroads database. With currently over 800 cataloged genes, this web-based resource constitutes the most extensive curation of genes relevant to HD. It provides us with an unprecedented opportunity to survey molecular mechanisms involved in HD in a holistic manner.

**Methods:**

To gain a synoptic view of therapeutic targets for HD, we have carried out a variety of bioinformatical and statistical analyses to scrutinize the functional association of genes curated in the HD Research Crossroads database. In particular, enrichment analyses were performed with respect to Gene Ontology categories, KEGG signaling pathways, and Pfam protein families. For selected processes, we also analyzed differential expression, using published microarray data. Additionally, we generated a candidate set of novel genetic modifiers of HD by combining information from the HD Research Crossroads database with previous genome-wide linkage studies.

**Results:**

Our analyses led to a comprehensive identification of molecular mechanisms associated with HD. Remarkably, we not only recovered processes and pathways, which have frequently been linked to HD (such as cytotoxicity, apoptosis, and calcium signaling), but also found strong indications for other potentially disease-relevant mechanisms that have been less intensively studied in the context of HD (such as the cell cycle and RNA splicing, as well as Wnt and ErbB signaling). For follow-up studies, we provide a regularly updated compendium of molecular mechanism, that are associated with HD, at http://hdtt.sysbiolab.eu Additionally, we derived a candidate set of 24 novel genetic modifiers, including histone deacetylase 3 (HDAC3), metabotropic glutamate receptor 1 (GRM1), CDK5 regulatory subunit 2 (CDK5R2), and coactivator 1ß of the peroxisome proliferator-activated receptor gamma (PPARGC1B).

**Conclusions:**

The results of our study give us an intriguing picture of the molecular complexity of HD. Our analyses can be seen as a first step towards a comprehensive list of biological processes, molecular functions, and pathways involved in HD, and may provide a basis for the development of more holistic disease models and new therapeutics.

## Background

Huntington disease (HD) is an autosomal-dominant neurodegenerative disorder characterized by severe impediment in movement, decline in cognitive functions, as well as behavioral abnormalities. In most cases, the first symptoms of HD are observed in patients between the ages of 35 and 50. The average life expectancy after disease onset is around 20 years. So far, no cure has been found for HD, and its treatment remains symptomatic. The prevalence of HD is highest for people with Caucasian descent with an estimated 40–100 cases per million. Notably, HD displays a distinct neuropathology: the disease primarily affects GABAergic medium spiny striatal neurons, leading to cell loss and atrophy of the caudate nucleus and putamen in the basal ganglia. Although the most prominent neuronal cell death is observed in the basal ganglia, other brain regions may show damage as well [1,2]. HD is caused by CAG repeat expansion in exon 1 of the *huntingtin* gene (identified in 1993 at chromosomal location 4p16 and originally termed IT15), which results in a long stretch of polyglutamine close to the amino-terminus of the Huntingtin protein (Htt) [3]. Remarkably, the length of the polyglutamine tract in the mutant Htt explains 40-50% of the variance in the age of onset, with longer repeats leading to earlier onset [4-8]. Although the exact mechanisms are yet to be understood, it is believed that HD results from both a gain-of-function of the mutant protein, as well as a loss-of-function of the wild-type protein [9]. Wild-type Htt is a large protein, over 3000 amino acids long, and is expressed ubiquitously throughout the body. Its sequence exhibits several consensus sites for protein interaction and modification, but it has no close homolog among human proteins based on its full sequence. Although wild-type Htt has been associated with several cellular processes, such as transcriptional regulation, vesicular transport and apoptosis; its exact function is not yet clear [10]. A distinct hallmark of HD is the appearance of cellular inclusion bodies due to the aggregation of mutant Htt.

Even though HD is caused by a mutation in a single gene, numerous additional genes may play a role in HD. Indeed, the observed variability in the pathogenesis, i.e. onset, severity and progression of HD, strongly indicates that other modifying environmental and genetic factors exist, apart from the disease causing poly-Q expansion in mutant Htt [3,11]. In particular, age of onset and disease symptoms can differ considerably between HD patients despite similar glutamine repeat length. Moreover, the residual variance in age of onset, i.e. the variance after adjusting for the differences in poly-Q length, shows a high degree of heritability [11]. These observations have evoked an intense search for genetic modifiers. As a result, several linkage studies have indicated that polymorphisms in other genes - apart from Htt - may modify onset and course of HD [12]. Furthermore, the use of RNAi screening in lower model organisms revealed that altering the expression of a surprisingly large number of genes can influence the aggregation of mutant Htt [13,14].

To systematically catalog the various potential genetic modifiers, as well as other genes influencing HD pathogenesis, CHDI Foundation has established a database termed HD Research Crossroads (or in short, HD Crossroads). This gene-centric resource currently summarizes information and validated data for over 800 genes relevant to HD pathogenesis. It provides links to supporting studies and presents the collected evidence of the role of these genes in HD. Thus, it enables the user to critically assess the classification of individual genes in HD Crossroads and thus their relevance to HD. (We will henceforth refer to the set of curated genes in HD Crossroads collectively as “HD-relevant” genes, although the amount of evidence for individual genes can be very different). Furthermore, HD Crossroads includes several scoring schemes for genes with respect to their potential as drug targets and allows registered users to add comments regarding particular genes. Its primary aim is to support the process of prioritizing gene targets for therapeutic intervention.

Apart from the gene-centric focus, the collected information provides a unique possibility for more holistic bioinformatical and statistical analyses. Since HD Crossroads comprises the largest and most comprehensive collection of curated HD-relevant genes to date, it can serve as a basis to obtain an overview of the biological mechanisms underlying HD. Such an assessment is not only important for HD research, but may be beneficial for the study of related diseases. Notably, HD is the most prominent example of the family of trinucleotide repeat disorders (including spinocerebellar ataxias 1, 2, 3, 6, 7, 17, spinobulbar muscular atrophy and dentatorubropallidoluysian atrophy), which share disease-related mechanisms despite their distinct phenotypes [15]. Thus, new insights into HD might give us a new rationale for the study of neurodegeneration in general.

Seeking a broader understanding of the molecular manifestation of HD, we utilized the curated set of HD-relevant genes and carried out extensive bioinformatical and statistical analyses. We translated the set of genes into a functional profile, exploiting annotation from several public databases. Our aim was to obtain a synoptic view of molecular processes and pathways that are enriched in HD-relevant genes. With these analyses, we have generated a compendium of molecular mechanisms that might play critical roles in HD. The compendium links biological processes, molecular functions and pathways to sets of HD-relevant genes and provides the research community with structured gene lists, for potential follow-up studies. Additionally, we have derived a list of candidates for genetic modifiers by integrating results from previous genome-wide linkage studies.

We would like to emphasize that this work is not intended as a review of the molecular pathogenesis underlying HD, although we will discuss our results with respect to numerous earlier findings. For a more exhaustive presentation of the current understanding of HD, the reader is referred to dedicated recent reviews [9,16,17].

## Methods

### HD Research crossroads database

The HD Research Crossroads database can be accessed at *http://www.hdresearchcrossroads.org/*. On user registration, the full list of curated genes can be downloaded from HD Crossroads. The genes included in the database are evaluated based on a variety of parameters such as Target Validation Score, Drugability Score, desired pharmacology, mechanistic class and target class. These parameters provide assistance in prioritizing target genes for pharmacological intervention. Notably, the collection of information and the inclusion of genes as targets is an on-going process, i.e., the HD Research Crossroads database is constantly being updated with the latest findings.

The most important parameter for our study is the Target Validation Score (TVS). The TVS of a gene captures the status of experimental validation of disease-modifying effects; it ranges from 0.0 to 5.0 (with 0.0 being lowest and 5.0 being highest). The scheme for assigning a TVS is as follows: a score of 5.0 is assigned to genes if a drug or a gene therapy modulating the gene has demonstrated efficacy in a Phase 3 clinical trial. A score of 4.5 is assigned to genes if a Phase 2 clinical trial of a drug or gene therapy had a positive outcome, or the HD phenotype in a non-rodent large animal mammalian model for HD (e.g. primate or sheep) is improved upon manipulation of the gene. A score of 4.0 signifies that an improvement of the HD phenotype in rodent models was observed using a therapeutically relevant drug or genetic intervention that is highly specific for this gene. A score of 3.5 is assigned when the gene is associated with HD in a linkage study in humans, or when the manipulation of the gene leads to changes in the HD phenotype in rodent models. A score of 3.0 is assigned to cases where a causal relationship with HD was observed in an *in vitro* cell culture or lower organism model of HD upon genetic or pharmacologic modification. A score of 2.5 is assigned to genes that show an altered pathway or functional activity in HD. A score of 2.0 is assigned if a gene shows a change in expression or cellular distribution in HD, or if the corresponding protein binds to mutant Htt. A score of 1.0 is assigned to genes present and active in HD-relevant brain regions or linked to a HD-relevant biological mechanism. Finally, a score of 0.0 is recorded for genes implicated in neurodegeneration or polyglutamine dysfunction based on genome-wide screens. Our analyses were carried out on the list of HD-relevant genes downloaded on 3^rd^ of January 2011.

### Functional enrichment analysis

All statistical analyses were performed in the R/Bioconductor environment (http://www.bioconductor.org; Biobase version 2.6.1) using several annotation packages. Different types of statistical investigations were conducted using different publically available resources. First, we carried out Gene Ontology (GO) based analyses. GO represents a structured, hierarchical system, which associates a gene product with a defined set of molecular functions, biological processes and cellular components. Currently, GO includes annotations for over 17,500 human gene products. In addition to the full set of GO categories, a reduced set of GO categories was utilized. It was created by the GO Consortium and is referred to as generic GO Slim. It includes only 127 categories (a subset of the 8245 categories of the full GO ontology) and can be downloaded from the GO website (http://www.geneontology.org/).

Our second analysis aimed to identify canonical pathways enriched in HD-relevant genes. For this purpose, we utilized information from the KEGG pathway database (http://www.genome.jp/kegg/pathway.html), which provides a large collection of manually derived schemes of metabolic and signaling pathways, as well as of a variety of diseases and other processes. The KEGG database employed in our analysis comprises over 5,000 human protein genes mapped to 220 pathways. Additionally, information from the Pfam database was used in order to examine whether specific protein families are over-represented in the set of HD-relevant genes. The Pfam database (http://pfam.sanger.ac.uk) is a collection of conserved protein families determined by multiple alignments and profiling through statistical models. Information from GO, KEGG and PFAM was obtained through their corresponding Bioconductor annotation packages, i.e., GO.db (version 2.3.5), KEGG.db (version 2.3.5) and PFAM.db (version 2.3.5).

Enrichment of HD-relevant genes in GO categories, KEGG pathways and Pfam protein families was tested using the Fisher's exact test. As reference set, the annotated human genome was used. Subsequently, statistical significance was adjusted for multiple testing, applying the Benjamini and Hochberg procedure [18]. The final significance for enrichment is given as False Discovery Rates (FDRs). The complete set of GO categories, KEGG pathways and Pfam protein families with their corresponding significance can be found in the supplementary materials. Since HD Crossroads is constantly expanding, we will to regularly repeat the enrichment analyses of HD-relevant genes and to provide these results on our webpage (http://hdtt.sysbiolab.eu/).

### Gene expression analysis

For gene expression analysis, microarray data for the human HD caudate nucleus and different HD mouse models (R6/2; BDNF-KO; CHL2; striatal cell line) were utilized [19-22]. Expression data were obtained from Gene Expression Omnibus database (GSE3790, GSE10263, GSE3583) and pre-processed using RMA (Robust Multi-array Average) as implemented in the R/Bioconductor environment [23]. The Bioconductor limma package was used to detect differential expression in HD compared with their corresponding controls. An adjusted p-value of 0.01 was selected as the threshold for significance. Additionally, a minimum absolute fold change of 1.2 was required for differential expression. Subsequently, the enrichment of the GO cell cycle and RNA splicing categories, as well as KEGG ErbB and Wnt pathways, by differentially expressed genes was calculated using Fisher's exact test. For promoter analysis, the Expander software with default settings was employed.

### Chromosomal mapping

The chromosomal location of genes was obtained from the Ensmart server (http://www.ensembl.org/Multi/martview) As loci modifying age of onset, we used those that were reported in the genome-wide scans by Li *et al.* (HD MAPS study) and by Gayán *et al.*[24,25]. The LOD score (logarithm (base 10) of odds) for suggestive evidence for linkage were retrieved from the publications. In the case of the HD MAPS study, the modifier loci and corresponding maximum LOD scores were given as 2q33 (LOD: 1.63), 4p16 (1.93), 5q31-32 (2.02), 6p22 (2.29), 6q23-24 (2.28) and 18q22 (1.74). In the case of the study by Gayán *et al*., the reported loci were 2p25 (4.29), 2q35 (3.39), 4p16 (1.49), 4p21 (2.07), 5p14 (3.31), 5q32 (3.14), 6q22 (2.48), 12q15 (2.86) and 19p13 (1.79).

## Results

### The HD Research Crossroads database and its scoring schemes

At present, the HD Research Crossroads database contains information about approximately 800 genes, for which a direct or indirect relevance to HD pathophysiology is indicated by available evidence. These genes might therefore be considered as targets for the development of therapies, which is a prime aim of HD research. The target information is gathered by evaluation of published studies and in-house screens.

**Figure 1:**
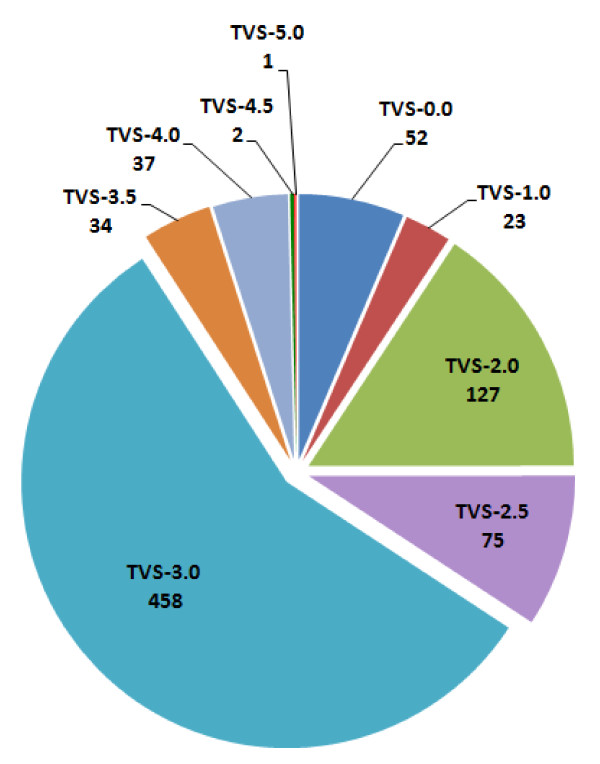
**Distribution of TVS. **The pie-chart shows the distribution of the Target Validation Score (TVS) in HD Crossroads. The numbers of genes with specific scores also are displayed.

To obtain an overview of the current state of curation by HD Crossroads, the distribution of TVS was visualized (Figure 1). It is evident that the majority of genes have a score of 3.0, which is assigned to a gene in the case that a causal relationship with HD was shown *in vitro* in cell culture or *in vivo* in a lower (non-rodent) model. In contrast, a TVS larger than 3.0 requires evidence from higher disease models or from linkage studies. Thus, it is not surprising that the number of genes with high scores is considerably smaller. At present, only SLC18A2 (VMAT2), a vesicular monoamine transporter, has a TVS of 5.0. Its inhibition by tetrabenazine, the first FDA-approved drug for treating HD-associated symptoms, results in decreased synaptic release of dopamine [26]. Further high scoring genes (TVS = 4.5) are: GRIN1, encoding a sub-unit of the glutamate receptor; and CNTF, encoding the ciliary neurotrophic factor. The Htt gene itself has a TVS of 4.0, as knock-down of its mutant form by RNAi was neuroprotective in rodent models of HD [27]. 

For our analyses, we utilized the TVS to filter the list of HD-relevant genes. We excluded genes with a TVS lower than 3.0, since the current evidence for a causal relationship of these genes with HD is limited. This step resulted in a list of 532 genes, for which a causal relationship to HD is indicated. An additional 162 genes with an updated TVS ≥ 3.0 were obtained directly from the database curators. The total list of 694 HD-relevant genes used for analyses and their corresponding TVS can be found in the Additional file 1.

### Functional composition of HD-relevant genes

For systematic evaluation of the filtered set of HD-relevant genes, we utilized information from the GO database. In GO, genes are annotated using a fixed vocabulary for the description of (i) biological processes (BP), in which a gene product is involved; (ii) molecular functions (MF), which it executes; and (iii) cellular compartments (CC), in which it is located. The GO vocabulary itself comprises more than 8 000 explicitly defined terms and relations between them. For our purpose, we utilized GO annotation for two tasks: firstly, we obtained the overall composition of HD-relevant genes; and secondly, we calculated the statistical significance of enrichment in HD-relevant genes for each GO category (i.e. the number of HD-relevant genes included in a GO category was compared with the number expected by chance).

**Figure 2:**
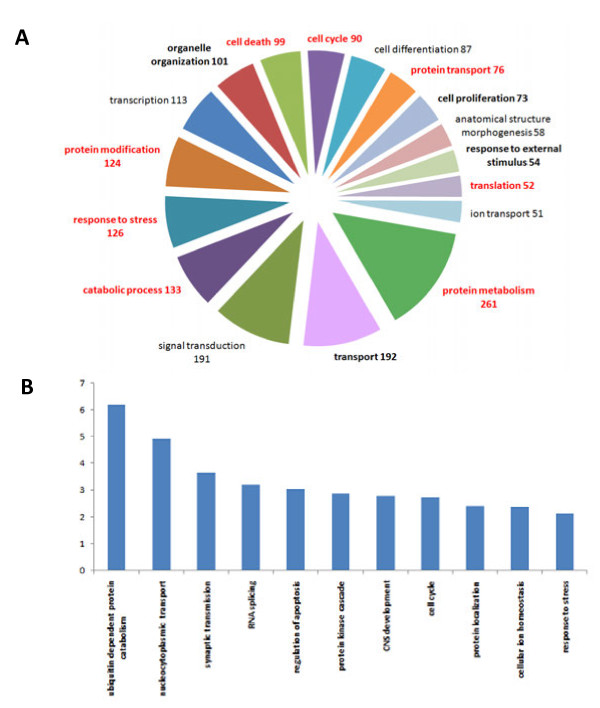
**Distribution of HD-relevant genes across biological processes. A**) Using GO annotation, HD-relevant genes were assigned to a reduced set of GO Slim categories, representing biological processes. Only processes with more than 50 HD-relevant genes are displayed on the pie-chart. Numbers refer to assigned HD-relevant genes. Biological processes that are significantly enriched (FDR ≤ 0.01) are set in bold. Red labels indicate an odds ratio ≥ 2.0 with respect to the number of genes expected by chance. Note that the BP categories are not exclusive, i.e., a gene can be assigned to several BP categories. **B**) Enrichment of HD-relevant genes displayed as odds ratios for selected biological processes from the full list of GO categories (see Additional file 2).

To facilitate interpretation of the functional composition, we restricted the number of GO BP categories. Initially, we only used terms in GO Slim, which comprises 127 categories, for main biological processes. Nevertheless, a direct interpretation was difficult due to the hierarchical structure of GO categories. For example, when a gene is ascribed to a category, it is automatically also associated with all of the relevant parent categories. Thus, a gene placed in the BP category “cell death” will also be associated with the parent category “death”, resulting in many dependent categories. To cope with this data structure, we excluded categories, when the majority of genes were associated with a single smaller category. Additionally, we required that a minimum number of 25 genes were included in a category. These filtering steps reduced the number of biological processes to 30. Figure 2A displays the distribution of HD-relevant genes across the reduced set of BP categories.

Remarkably, a diverse set of processes was obtained; with protein metabolism (38% of HD-relevant genes), transport (28%), signal transduction (28%), catabolic process (19%), and response to stress (18%) as the five largest categories. Note that GO categories are not exclusive, and genes or gene products can be associated with multiple processes. The full list of BP categories, together with corresponding HD-relevant genes, can be found in the supplementary materials (Additional file 2).

**Figure 3:**
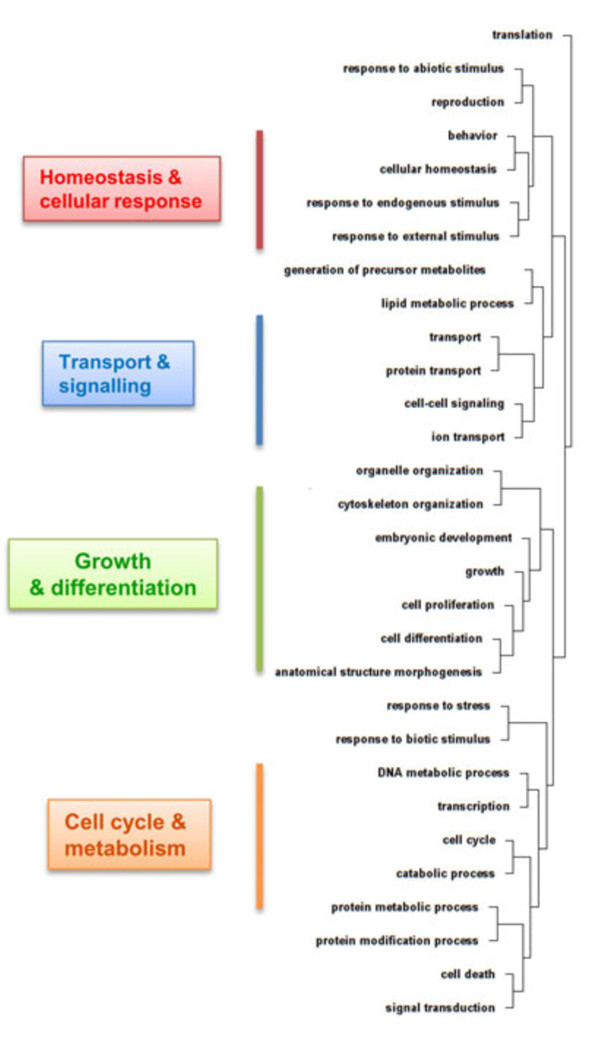
**Hierarchical clustering of biological processes. **HD-relevant genes were assigned to biological processes. Similarity of biological processes was defined by the percentage of shared genes. Based on the defined similarity, subsequent hierarchical clustering of biological processes was carried out.

Although we reduced the list of GO categories, their evaluation remained challenging, since they are not mutually exclusive and genes can be associated with multiple GO categories. In order to facilitate our examination and to obtain a more structured functional profile, we carried out hierarchical clustering of GO BP categories. The relative overlap in HD-relevant genes was used as a similarity measure, so that categories sharing HD-relevant genes tended to cluster. The dendrogram obtained (Figure 3) displays several larger clusters, encapsulating processes related to homeostasis and cellular response, transport and signaling, growth and differentiation, as well as cell cycle and metabolism. Out of these four cardinal groups, two (i.e. homeostasis and cellular response, transport and signaling) can be tentatively linked to excitotoxicity, which was one of the first mechanisms proposed to explain the tissue-specific damage in HD [28]. Excitotoxicity is a pathological process, resulting in neuronal cell death caused by an over-activation of glutamate receptors. It may contribute to several neurodegenerative disorders, as well as other brain injuries. N-Methyl-D-aspartic acid (NMDA) and kainic acid (as well as glutamate itself at high levels) are prime examples of excitotoxins, leading to an excessive influx of calcium ions. A resulting failure of cellular calcium homeostasis causes mitochondrial and membrane damage, as well as the generation of free radicals and might eventually trigger neuronal apoptosis. 

### Biological processes overrepresented among HD-relevant genes

To evaluate the statistical significance of the observed broad distribution, we subsequently performed an enrichment analysis. Again, employing the full set of GO categories, we found a strikingly large number of categories enriched in HD-relevant genes. In total, 212 categories with 25 or more HD-relevant genes yielded a false discovery rate (FDR) of less than 0.01 for enrichment (Additional file 2). Inspection of categories enriched in HD-relevant genes identified processes that are specific to the central nervous system such as neuron development (26 genes; FDR = 7 · 10^-4^) and synaptic transmission (40 genes; FDR = 6 · 10^-9^). However, the majority of categories enriched in HD-relevant genes were made up by generic cellular processes, many of which have been previously linked to HD (Figure 2B). For example, one of the most significantly enriched processes is ubiquitin-dependent proteasome degradation (50 genes; FDR = 4 ·10^-18^). This reflects the substantial evidence that the ubiquitin-proteasome system, which is crucial for degrading and removing misfolded and damaged proteins, becomes dysfunctional due to congestion by mutant Htt [29-32]. Indeed, one of the main hallmarks of HD neuropathology is the appearance of intracellular ubiquitin-positive inclusion bodies of mutant Htt [30]. Such anomalous protein aggregation might be caused by an impaired ubiquitin-proteosome system, as observed in the striatum and the cortex of human HD patients [33]. Other enriched processes, previously connected to pathogenesis of HD, include apoptosis (92 genes; FDR = 5 · 10^-12^) [34-37], intracellular-vesicular transport (71 genes; FDR = 2 · 10^-10^) [38-40] and cellular ion homeostasis (30 genes; FDR = 8 · 10^-4^) [41,42].

**Figure 4:**
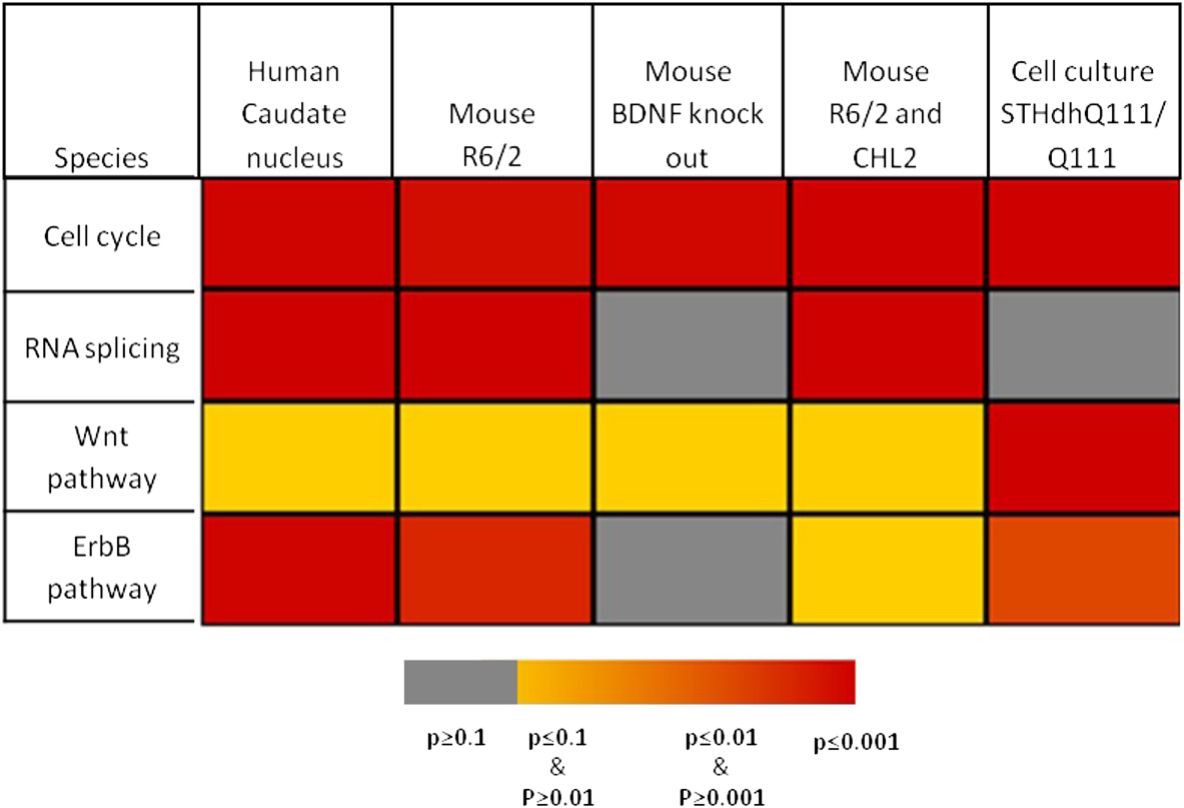
**Significance of enrichment in differentially expressed genes for human HD samples and disease models. **The heat-map depicts the significance of enrichment in differentially expressed genes for GO process cell cycle and RNA splicing, as well as for the KEGG Wnt and ErbB pathway. P-values were derived by Fisher’s exact test.

Further examination of the results revealed highly significant enrichment of a number of biological processes, for which the molecular details of their connection with HD are not yet fully elucidated. For instance, RNA splicing was highly enriched in HD-relevant genes (37 genes; FDR = 4 · 10^-7^). To explore the possible connection of RNA splicing to HD, we examined the experimental evidence which supported the listing of corresponding genes in HD Crossroads. We found that two RNAi screens for modifiers of aggregation in *C. elegans* and *Drosophila* cells were contributing the vast majority of genes (N = 33 genes) [13,43]. To determine whether RNA splicing could also play a role in rodent HD models and human patients, we re-analyzed several publically available microarray data sets (Figure 4). Strikingly, we found that genes related to RNA splicing tend to be differentially expressed in human HD samples compared with controls (p = 1 · 10^-4^), as well as in R6/2 mice compared with wild-type (p = 3 · 10^-4^). This suggests a potential dysregulation of the splicing machinery in HD. To explain the influence of RNA splicing genes on aggregation, it has been proposed that the disruption of RNA splicing interferes with protein homeostasis and enhances aggregation by a general increase of misfolded proteins [13]. Alternatively, RNA splicing might be directly affected by distorted physical interaction with mutant Htt. Here, work by Marcy MacDonald and colleagues has provided some important cues [44,45]. First, they demonstrated that PRPF40A and PRPF40B, human homologs of the yeast splicing factor pre-mRNA processing protein 40, interact via WW domains with the N-terminal proline-rich segment of Htt [44]. In a follow-up study, PRPF40A was also found to be recruited to inclusion bodies. More interestingly, both pre-RNA splicing factors exhibited exaggerated binding to mutant Htt [45]. Such aberrant interaction appears to interfere with the efficiency of RNA splicing [46]. 

Intriguingly, another GO process highly enriched in HD-relevant genes is the cell cycle (90 genes; FDR = 3 · 10^-12^). This is somewhat surprising, since the cell types mainly affected by HD are differentiated neurons that are typically seen as permanently post-mitotic. In contrast to the case for RNA splicing, the significance of enrichment in this case is not due to a small number of studies, but derives from integration of results from over 40 publications. We verified that the significance of the cell cycle is not simply caused by a large overlap with other distinct categories overrepresented among HD-relevant genes. This was however not the case. GO terms with large overlap were either cell-cycle related such as “mitotic cell cycle” (64 genes) or rather of a generic nature such as “regulation of molecular function” (47 genes) or “regulation of catalytic activity” (46 genes). To obtain further support for a connection between HD and the cell cycle, we again utilized genome-wide expression data. Remarkably, cell cycle genes tend to be differentially expressed in human HD samples (p = 2 · 10^-4^), HD mouse models (R6/2: p = 6 · 10^-4^; BDNF-KO: p = 4 · 10^-4^), as well as a murine striatal cell line (p = 1 · 10^-4^). Promoter analysis of HD-relevant genes also supported these observations. We detected a highly significant enrichment in binding sites for the E2F transcription factor family, which is crucial for cell cycle control, upstream of HD-relevant genes (p < 10^-15^). It should be noted, that results from expression data need to be treated with caution. Except for the striatal cell line, samples from human HD patients and mouse models can be expected to differ considerably in the composition of cell types from their corresponding controls, since neurons are generally more vulnerable to mutant Htt than glia cells. This might especially impact on the expression of cell cycle genes, as glia cells are still mitotic. This difficulty in the interpretation of HD microarray data has been previously assessed in a study by Hodges *et al*., in which laser-capture microdissected neurons were profiled and compared with expression data from homogenate samples for a small number of brains [19]. In general, the expression changes observed in both data sets displayed significant correlation. However, further study is warranted regarding this issue.

So far, studies connecting HD with the cell cycle process are sparse. One notable connection was the observed increase in cell proliferation and neurogenesis in the adult human HD brain [47]. Examination of postmortem tissue showed that the degree of cell proliferation in the subependymal zone adjacent to the caudate nucleus increased with pathological grade and length of the Htt polyglutamine repeat. This finding was reproduced in mouse models of HD, although neurogenesis might decrease in other parts of the brain during disease progression [48-50]. An even more direct link between the cell cycle and HD may be provided by the intriguing possibility that re-entering the cell cycle leads to neurodegeneration and cell death of neurons. Although mature neurons are normally seen as post-mitotic, several studies indicate that they ectopically re-express numerous cell-cycle markers and replicate their DNA in various neurodegenerative disorders, including Alzheimer’s and Parkinson's disease [51]. These observations are in line with experiments where blocking the cell cycle by inhibiting cyclin-dependent kinases protects against neuronal death [52,53]. To our knowledge, this potential connection between cell cycle and neurodegeneration has not been assessed yet for HD.

### Cellular location of HD-relevant gene products

**Figure 5:**
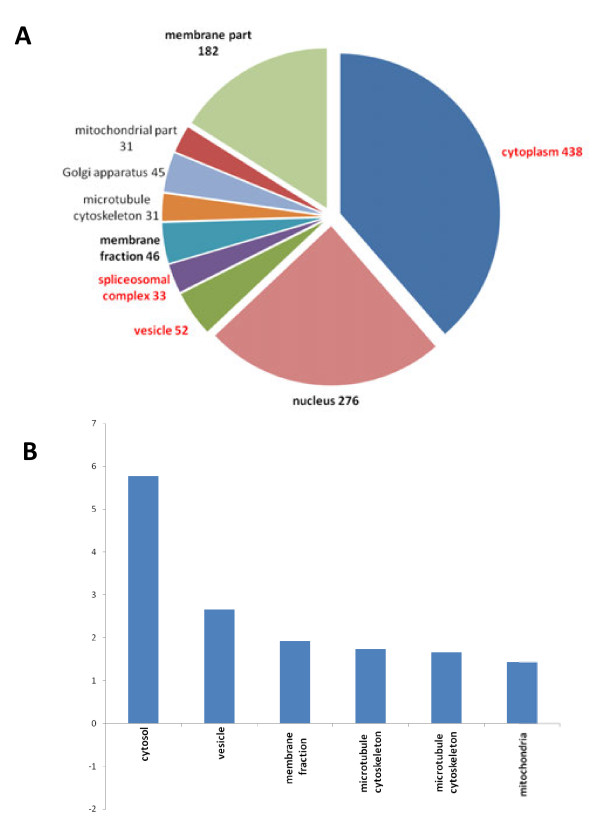
**Distribution of HD-relevant genes across cellular components. A**) The pie-chart shows the distribution and the number of HD-relevant gene products allocated to GO Slim categories for cellular components. GO terms that are significant (FDR ≤ 0.01) are set in bold and terms with an odds ratio ≥ 2.0 are set in red. Note that the cellular component categories are not exclusive, i.e., a gene can be assigned to several categories. **B**) The plot displays odd ratios for enrichment of selected cellular components.

Next, we surveyed the cellular location of the products of HD-relevant genes based on their GO Slim annotation. We observed that they are widely distributed across different compartments (Figure 5). The majority of HD-relevant gene products are located in the cytoplasm (63%), of which about a third are harbored in the cytosol (i.e. the cytoplasm excluding organelles). Approximately 40% of gene products can be assigned to the nucleus and a further 25% to the membrane part of the cell. The statistical enrichment analysis yielded a high significance for major cellular compartments: cytoplasm (438 genes; FDR = 5 · 10^-55^), nucleus (276 genes; FDR = 4 · 10^-10^) and membrane part (182 genes; FDR = 2 · 10^-4^) (Additional file 3). Additionally, the analysis also revealed a significant enrichment in HD-relevant gene products for organelles such as vesicles (52 genes; FDR = 8 · 10^-8^) and Golgi apparatus (45 genes; FDR = 0.05), as well as molecular structures such as microtubules (31 genes; FDR = 0.03) and ribosomes (31 genes; FDR = 1 · 10^-9^). We also detected the spliceosomal complex as highly enriched (33 genes; FDR = 3 · 10^-16^), which is in concordance with the previously noted significance of RNA splicing. In contrast, mitochondria did not reach significance (46 genes; FDR = 0.33), although mitochondrial alterations may play an important role in HD pathogenesis. Notably, the mitochondrial envelope treated as a separate sub-compartment was detected as enriched in HD-relevant gene products (26 genes; FDR = 0.02). This agrees with results of previous studies exploring mitochondrial changes due to mutant Htt in both cell culture and animal models [54,55]. They showed that N-terminal fragments of mutant Htt associate with the outer membrane of mitochondria and interfere with trafficking of mitochondria, as well as with mitochondrial membrane permeability; lowering the threshold for calcium-induced release of cytochrome c. 

In general, the distribution of HD-relevant genes seems to reflect the widespread subcellular location of the different products of Htt gene products: wild-type Htt is primarily a cytoplasmic protein associated with a variety of cellular structures and organelles such as microtubules, vesicles, Golgi complex, endoplasmic reticulum and axon terminals of neurons; whereas fragments of mutant Htt can also be found in inclusion bodies in the nucleus [56-58].

### Molecular functions of HD-relevant genes

We also evaluated the molecular functions of HD-relevant genes based on their GO Slim annotations (Additional files 4 and 5). The most common molecular functions of HD-relevant genes were generic activities: protein binding (73%; N = 509 genes) and catalysis (42%; N = 293 genes). Both categories were also highly significant with FDR = 5 · 10^-45^ and FDR = 3 · 10^-14^. More specific functional categories overrepresented among HD-relevant genes comprised transporters (70 genes; FDR = 0.02), structural molecules (54 genes; FDR = 7 · 10^-6^) and protein kinases (50 genes; FDR = 3 · 10^-5^). In addition to GO annotation, we employed information from the Pfam database enabling the classification of HD-relevant genes into protein families (Additional file 6). The largest and most significantly enriched Pfam family is protein kinase (41 genes; FDR = 10^-6^), which matches with the results from the GO analysis. Although the database coverage by Pfam seems to be lower than by GO, significance in enrichment was obtained for various protein families with functions previously linked to HD. For example, we found that members of the ligand-gated ion channel (5 genes; FDR = 5 · 10^-5^), histone deacetylase (6 genes; FDR = 2 · 10^-6^), caspase (5 genes; FDR = 10^-4^) and ubiquitin (8 genes; FDR = 10^-4^) family tend to be HD-relevant. Notably, HD-relevant genes displayed enrichment in several different families of chaperons: Hsp20/alpha crystallin family (6 genes; FDR = 3 · 10^-7^), DnaJ domain family (10 genes; FDR = 10^-4^), Hsp70s (5 genes; FDR = 10^-4^), and TCP-1/cpn60 chaperonin family (6 genes; FDR = 10^-4^); which play important roles in protein folding and aggregation, as well as assembly and disassembly of macromolecular structures.

### KEGG pathway analysis

Signaling pathways are of central importance for the regulation and coordination of cellular events. Their aberrant activation is often linked to the development of major diseases. Considerable research has been carried out to identify components of cellular signaling, their sequential activation or deactivation, as well as their interactions. Notably, numerous drugs have been established to alter the function of relevant pathways. Although their full complexity is still far from being completely understood, signaling pathways – or rather the current canonical models of them – can offer a conceptual framework for the development of pharmacological intervention.

To evaluate whether specific signaling pathways might be implicated in HD or offer therapeutic targets, we utilized the canonical models as defined in the KEGG pathways database. It is however important to keep in mind that the pathway models are not exclusive, i.e., they show considerable overlap, reflecting the frequently observed synergy in signaling. Also, statistical significance of enrichment in HD-relevant genes does not necessarily signify that the pathway plays a role in the actual HD pathogenesis, but rather indicates that the pathway can represent an effective target for intervention.

**Figure 6:**
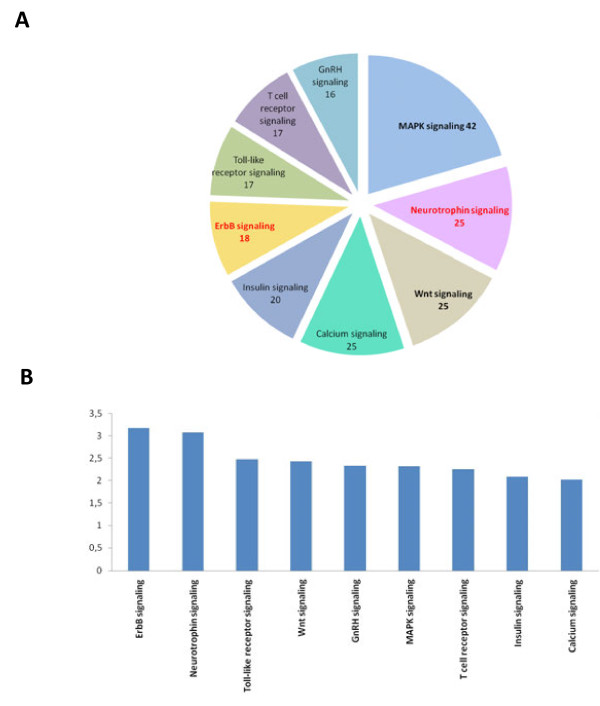
**Association with major KEGG pathways. A**) KEGG signaling pathways with more than 15 HD-relevant genes and an odds ratio ≥ 2.0 are included on the pie-chart. Numbers refer to HD-relevant genes in a pathway. Highly significantly enriched pathways (FDR ≤ 0.01) are highlighted by bold font. Pathways with odds ratio ≥ 3.0 are set in red font. **B**) Odd ratios are displayed for enrichment of selected pathways.

Besides signaling pathways, KEGG comprises models for a variety of processes and molecular complexes. The mapping of the HD-relevant genes onto these models revealed that processes such as apoptosis (21 genes; FDR = 8·10^-5^), ubiquitin mediated proteolysis (23 genes; FDR = 2·10^-4^) and endocytosis (22 genes; FDR = 0.08), as well as protein complexes such as ribosome (31 genes; FDR = 7·10^-12^), proteasome (21 genes; FDR = 7·10^-10^) and gap junctions (16 genes; FDR = 1·10^-4^) tend to accumulate HD-relevant gene products (Additional file 7). We observed that 13 signaling pathways were enriched (FDR < 0.05). Figure 6 displays the distribution and odd ratios of the major pathways with a minimum of 15 HD-relevant genes included. The highest significance in enrichment was obtained for MAPKK (42 genes; FDR = 2·10^-4^), neurotrophin (25 genes; FDR = 2·10^-4^), ErbB (18 genes; FDR = 1·10^-3^), and Wnt signaling (25 genes; FDR = 2·10^-3^).

For several of these pathways, such as MAPK, mTOR, insulin and calcium ion signaling, a role in HD had previously been proposed (see Table 1). Interestingly, we also detected significant enrichment in a number of pathways, which have received less attention for their role in HD. For instance, Toll-like receptor (TLR) signaling was found to be significantly enriched (17 genes; FDR = 10^-3^). Primarily known for their role in detecting pathogens and activating the immune response, TLRs have only recently been linked to neurodegeneration [59]. In mouse models of Alzheimer disease, loss-of-function mutation or knock-downs of TLRs were reported to increase extra-cellular Aβ deposits, as well as enhance cognitive deficits, which could be the result of activated microglia [60,61]. In HD, where protein aggregation produces intra-cellular inclusion bodies, a more relevant process might be the activation of autophagy by TLR via myeloid differentiation factor 88 (MyD88). Autophagy is a fundamental process that degrades cystosolic constituents, including unfolded or misfolded proteins and may have protective effects against neurodegeneration by degrading toxic cytosolic protein aggregates [62]. Thus, the link between TLR signaling and autophagy could be a valuable site for therapeutic intervention in treating HD, but its relevance for HD needs further evaluation, since TLRs are cell-type specifically expressed and carry out a divergent range of functions. 

**Table 1:** Signaling pathways with established links to HD

**Pathway**	**Evidence and suggested role in HD**	**HD-relevant genes**	**Significance (FDR)**
**Calcium ion signaling**	Mutant Htt affects calcium signaling in primary cultures of medium spiny neurons from HD mouse models by increasing the influx of extra-cellular Ca^2+^ and the intra-cellular release of Ca^2+^ from ER [63,64]	25	0.01
**MAPK signaling**	Induction of mutant Htt in PC12 cells leads to activation of MAPK signaling via ERK and JNK. ERK phosphorylation correlates with reduced apoptosis, whereas JNK activity correlates with Htt-associated cell death in a striatal HD cell line [65]	42	0.0002
**Insulin signaling**	The insulin signaling pathway promotes survival of striatal cells (transfected with mutant Htt) through neuroprotective effects of Akt activation and triggers autophagy mediated clearance of huntingtin via insulin receptor substrate-2 in HD cell model [66]	20	0.02
**mTOR signaling**	Inhibition of mTOR by rapamycin or by sequestration into aggregates induces autophagy in HD cell model and reduces neurodegeneration in HD fly and mouse model [62]	17	0.01

Signaling pathways that have been previously linked to HD progression and their corresponding significance of enrichment in HD-relevant genes.

The importance of the Wnt pathway (which was detected as enriched in HD-relevant genes) has been well established for developmental processes. Its potential role in neurodegeneration, however, is less well characterized [67]. In this context, previous research has focused on the study of glycogen synthase kinase (GSK)-3ß, which is a central component in the canonical Wnt pathway and suppresses its activity unless stimulated by the Wnt ligand. Notably, GSK-3ß is also an integral part of other pathways such as insulin signaling and thus emerges as a convergence point for different kinds of signals [68]. In Alzheimer's disease, GSK-3ß has been associated with hyperphosphorylation of tau protein and the formation of neurofibrillary tangles - a hallmark of Alzheimer's disease [67]. In addition to hyperphosphorylated tau, a decrease in nuclear ß-catenin (a transcriptional co-activator whose turnover is regulated by GSK-3ß phosphorylation) has been observed in transgenic mice overexpressing GSK-3ß in hippocampal neurons [69]. These changes were accompanied by increased neuronal cell death and astrocytosis in the hippocampus. Notably, blocking the activity of GSK-3 ß by lithium in transgenic mice reduced aggregation of tau and axonal degeneration [70]. For HD, several studies also point to alterations in Wnt signaling. In a mouse HD model, Gines *et al.* observed increased levels of activated Akt, which could be reproduced in a derived striatial neuronal cell line [71]. This change was associated with inhibition of GSK-3ß phosphorylation and stabilization of ß-catenin. The authors proposed that the activation of Akt can be seen as an early pro-survival response to enhanced NMDA receptor activity. Interestingly, an increase in expression of cyclin D1, a target gene of the ß-catenin/TCF transcription complex, was detected. This may be relevant for the previously indicated involvement of cell cycle genes, as ectopic cyclin D1 expression was also observed in sites of neuronal cell death in Alzheimer's disease patients [72]. Consistent with earlier studies, direct blocking of GSK-3ß by lithium treatment in the HD *Drosophila* model was protective against polyglutamine-mediated toxicity [70]. The protection was at least partially caused by the up-regulation of transcriptional target genes in the Wnt pathway, since reduced toxicity was observed for overexpression of TCF. Thus, impairment of Wnt signaling seems to contribute to neurodegeneration. In HD gene expression data, we found some indications for perturbation of the canonical Wnt signaling in human patients (p = 0.02), R/2 mice (p = 0.04) and in the HD striatal cell culture model (p = 0.0001). In conclusion, it appears that an involvement of Wnt signaling in neurodegeneration in general, and specifically in HD, was indicated by different lines of investigations. However, the molecular details of this connection require further scrutiny, as another recent study of the role of ß-catenin in HD indicates. In agreement with the study by Gines *et al.*, Humbert and co-workers have observed an accumulation of ß-catenin in different HD models, as well as post-mortem samples from patients [73]. The accumulation could be explained by an interference of mutant Htt with the degradation of ß-catenin. Importantly, the accumulated ß-catenin was phosphorylated, which normally predates ubiquitination and degradation of ß-catenin, and did not result in transcriptional activation. In apparent contrast to previous studies, this accumulation of ß-catenin appeared to be toxic, whereas its enhanced degradation reduced polygluatamine-increased neuronal toxicity.

Another pathway that was found to be significantly enriched in HD-relevant genes in our study is ErbB signaling. It is activated upon ligand binding to ErbB receptor kinases, which are also known as epidermal growth factor receptors (EGFRs). The pathway plays a key role in mediating intercellular signals during organogenesis; and aberrant expression of ErbB proteins and their ligands have been associated with various types of cancers [74,75]. For neuronal development, neuregulins (NRGs) as ligands of ErbBs are of importance. In particular, NRG1 and its receptor ErbB4 have been implicated in neuronal migration, axon guidance and synapse formation [76]. Notably, both proteins are expressed in the adult brain and modulate the properties of glutamatergic synapses through the binding of ErbB4 to postsynaptic density protein 95 (PSD95). Interestingly, PSD95 itself can bind to both NMDA receptors and Htt. In transfected cell lines, mutant Htt interferes with the ability of wild-type huntingtin to interact with PSD95 leading to the sensitization of NMDA receptors, thereby potentially promoting excitotoxicity [77]. The polyglutamine expansion in Htt might also have a direct effect on ErbB signaling and may antagonize the activation of down-stream targets. Such a mechanism is supported by the results of several studies. After demonstrating that wild-type Htt is associated with the EGFR signaling complex after stimulation [78], Liu and co-workers observed that EGF-stimulated phosphorylation of MAPK, AKT and JNK is strongly reduced in cells transfected with mutant Htt [79]. Their work also indicates that mutant Htt interferes with the assembly of the signaling complex associated with stimulated EGFR. These findings agree well with results from *in vivo* studies in flies [80]. Here, it was shown that co-expression of Htt exon 1 with expanded polyglutamine tract strongly inhibited EGFR-mediated MAPK phosphorylation in glia cells. Notably, changes in expression of the glutamate transporter could be reverted by activated MAPK, but not activated EGFR alone, indicating that polyglutamine acts on EGFR signaling upstream of MAPK. In our analysis, the genes associated with the ErbB signaling pathway tend to be dysregulated in humans (p = 2·10^-4^) and in the R6/2 mouse model (p =2·10^-3^). Finally, recent studies have linked perturbations in ErbB signaling to Alzheimer's disease, multiple sclerosis, and schizophrenia [81].

### Prediction of genetic modifiers by data integration strategy

Major efforts in HD research have been undertaken to identify genetic modifiers, i.e., genes with polymorphisms that alter HD pathogenesis. A main strategy has been genome-wide linkage analysis to detect genetic loci modifying the age of disease onset. In general, the determination of genetic factors influencing complex traits such as age of onset is notoriously difficult, as multiple factors with incomplete penetrance are likely to play a role. Despite considerable cost in resources, linkage analyses of pedigrees are a highly attractive paradigm, since (by definition) the identified genetic modifiers have demonstrated an effect in humans. In contrast, genome-wide screens for disease modifiers have become readily available in lower organisms, but detected modifiers still need to be validated for humans, presenting a major bottleneck for the development of novel therapies. So far, two genome-wide studies have been performed to find loci influencing age of onset of HD [24,25,82]. Notably, both studies detected multiple loci in distinct population samples. A limitation of these studies, however, is their coarse resolution, identifying rather long regions with a large number of genes. Hence, finding genetic modifiers remains challenging. The resolution can be expected to improve with the use of microarray technologies for genotyping, but this will also demand a considerably larger number of samples in order to ensure sufficient statistical power to detect associations [83].

We reasoned that the curated set of HD-relevant genes could be of complementary value for the identification of genetic modifiers. Since these genes have demonstrated a causal relationship, mostly in lower HD models, they are potential modifiers of HD, assuming the underlying mechanisms are conserved. If their human orthologs are additionally located in the regions linked to altered age of onset, these genes can be regarded as prime candidates for genetic modifiers of HD. Thus, we can narrow down the list of potential genetic modifiers found in the linkage studies by employing information from HD Crossroads.

**Figure 7:**
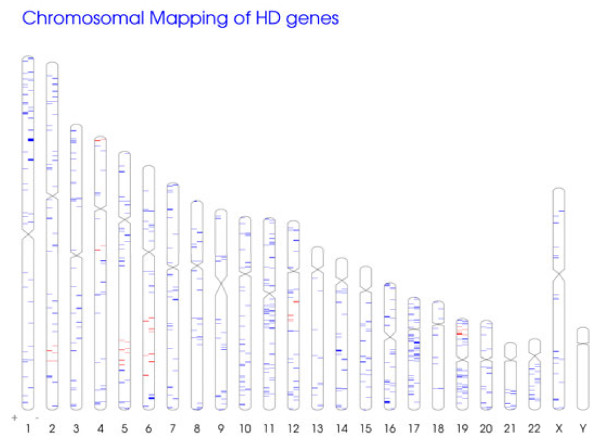
**Chromosomal location of HD-relevant genes. **Red marks highlight HD-relevant genes located in chromosomal regions that have been linked to the age of onset by genome-wide linkage studies. Blue marks represent HD-relevant genes outside these regions.

With this objective, we first mapped all HD-relevant genes to their loci on human chromosomes. The distribution across the human genome is displayed in Figure 7. Subsequently, we searched for chromosomal regions, for which potential linkage has been reported in both studies. We found that peaks in the LOD score were reported for locations in close vicinity in four distinct chromosomal regions: i) *2q33-2q35* with LOD_1_ =1.63 at 2q33 by Li *et al*. and LOD_2_ = 3.39 at 2q35 by Gáyan *et al.* ii) *4p16* with LOD_1_ = 1.93 and LOD_2_ = 1.49, iii) *5q31-32* with LOD_1_ = 2.02 at 5q31-32 and LOD_2_ = 3.14 at 5q32 and iv) *6q22-24* with LOD_1_ = 2.28 at 6q23-24 and LOD_2_ = 2.48 at 6q22 [24,25]. Although some of these peaks may not have reached genome-wide significance, the fact, that they were detected in close proximity by two independent studies supports the inclusion of the corresponding regions in our analysis. In total, 26 HD-relevant genes can be found in these loci. This reduces the total number of genes (N = 1270) located in the indicated regions by a factor of 50. The list of the HD-relevant genes and their loci is shown in Table 2. A complete listing of genes from HD-Crossroads, which are located in any of the chromosomal regions indicated by the genome-wide scans, can be found in Additional file 8. 

**Table 2:** Candidate list of genetic modifiers

	**Entrez ID**	**Symbol**	**TVS**	**Chr.**	**Band**
**Region = 2q33-2q35**	7341	SUMO1	3	2	q33.1
	9689	BZW1	3	2	q33.1
	23451	SF3B1	3	2	q33.1
**HD MAPS: LOD =1.63 (at 2q33)**	1385	CREB1	3.5	2	q33.3
**Gayan et al: LOD =3.39 (at 2q35)**	7855	FZD5	3	2	q33.3
	3300	DNAJB2	3	2	q35
	8941	CDK5R2	3	2	q35
	10109	ARPC2	3	2	q35
**Region = 4p16**	6286	S100P	3	4	p16.1
	9948	WDR1	3	4	p16.1
**HD MAPS: LOD =1.93**	118	ADD1	3	4	p16.3
**Gáyan et al : LOD =1.49**	3064	HTT	4	4	p16.3
	7469	WHSC2	3	4	p16.3
**Region = 5q31-32**	3308	HSPA4	3.5	5	q31.1
	6500	SKP1	3	5	q31.1
	2107	ETF1	3	5	q31.2
**HD MAPS: LOD = 2.02 (at 5q31-32)**	3313	HSPA9	3	5	q31.2
**Gáyan et al: LOD =3.14 (at 5q32)**	7322	UBE2D2	3	5	q31.2
	8841	HDAC3	4	5	q31.3
	10915	TCERG1	3	5	q32
	133522	PPARGC1B	3	5	q32
**Region = 6q22-24**	2444	FRK	3	6	q22.1
	6206	RPS12	3	6	q23.2
**HD MAPS: LOD =2.28 (at 6q23-24)**	9439	MED23	3	6	q23.2
**Gáyan et al: LOD =2.48 (at 6q22)**	4217	MAP3K5	4	6	q23.3
	2911	GRM1	3	6	q24.3

HD-relevant genes located in the four chromosomal regions implicated by both genome-wide scans [24,25]. The LOD scores reported in these studies for the exact location are shown on the left column.

Two genes (i.e. MAP3K5/ASK1 and TCERG1) from this list have already been proposed as genetic modifiers in HD based on previous association studies [84,85]. Genetic variation around the loci of mammalian mitogen-activated protein kinase kinase kinase 5 (MAP3K5), also termed apoptosis signal-regulating kinase 1 (ASK1), has been associated with modified age of onset [84]. ASK1 plays an important role in the poly-Q induced neuronal cell death and is activated by various stimuli. In particular, persistent ER stress and UPR activation triggered by extended polyQ repeats can activate ASK1, inducing apoptosis [86]. Another previously proposed modifier is the transcriptional activator CA150 (TCERG1) located at 5q32. It has been detected in inclusion bodies and has been shown to interact with full-length Htt *in vitro*[85]. TCERG1 contains a gluatamine-alanine repeat in its N-terminus; its length was negatively correlated with the age of onset [85,87]. Intriguingly, TCERG (besides its role as a transcriptional co-activator) has also been associated with the spliceosome and exhibits a domain composition similar to the Htt-interacting splicing factor PRPF40 (including both WW and FF domains) [88]. Thus, TCERG1 may provide a further connection between RNA splicing and HD. It should be noted, however, that the identification of these two potential genetic modifiers cannot serve to cross-validate our approach (but rather to illustrate), since the inclusion of at least one (MAP3K5) in HD Crossroads was based on its association with age of onset.

Among the other 24 genes identified, we were especially interested in those that have high TVS in HD Crossroads due to their potential as targets for novel therapies. The compiled list included three genes with a TVS = 4, indicating that the phenotype in rodent HD models was improved upon specific targeting of these genes. Besides ASK1 and Htt, histone deacetylase 3 (HDAC3) has a TVS = 4, since its inhibition by small molecules yielded a positive effect in the R6/2 mouse model [89]. A further two genes with TVS of four were found in the set of genes with a chromosomal location supported by only one study: cAMP-specific phosphodiesterase 4A (PDE4A) and neurturin (NRTN). Inhibition of PDE4A showed ameliorated HD phenotype in mice, where expression of neurotrophic factor NRTN was shown to be protective for striatal neurons against excitotoxicity induced by quinolinate injection [90,91].

The remaining HD-relevant genes in the selected regions are involved in wide range of processes, of which, some play important roles in HD. For instance, metabotropic glutamate receptor 1 (GRM1), a G-protein-coupled receptor with its gene located at 6q24, is a component of the glutamatergic neurotransmission apparatus and thus might be linked to excitotoxicity. Although the over-stimulation by glutamate is mainly inflicted through ionotropic NMDA receptors, studies have indicated that GRM1 modulates glutamatergic neurotransmission and eventually excitotoxicity [92]. Inhibition of GRM5, which belongs to the same sub-group of metabotropic glutamate receptors as GRM1, has already been demonstrated to attenuate disease progression in an HD mouse model [93]. Moreover, other genes involved in glutamatergic neurotransmission have previously been proposed to act as genetic modifiers of HD: GRIK2 encoding the kainite-type glutamate receptor; as well as GRIN2A and GRIN2B, encoding NMDA receptor sub-units [87].

Another gene which can readily be linked to a potentially disease-relevant mechanism is PPARGC1B. It activates various transcription factors and nuclear receptors such as the estrogen receptor alpha and is involved in the regulation of energy metabolism, which appears to be affected in HD. Remarkably, a recent study associated another member of the same gene family (i.e., PPARGC1A) with a delay in age of onset of motor symptoms in HD patients [94].

A gene which may also warrant closer inspection is CDK5R2, which encodes a neuron-specific activator (p39) of Cyclin-dependent kinase-5 (CDK5). In contrast to other cyclin-dependent kinases, CDK5 appears not to be directly involved in regulating progression through the cell cycle, but has been associated with neurogenesis, as well as with neurodegenerative disease [95,96]. Its localized activity in neurons can be attributed to its activators (p35 and p39). Interestingly, CDK5 is a proline-directed serine/threonine kinase with numerous substrates, one of which is Htt. It could be shown that phosphorylation of mutant Htt by CDK5 reduces its cleavage by caspases, as well as its toxicity [97].

As this brief review of the derived gene list suggests, an integrative strategy can deliver promising candidates for genetic modifiers associated with various disease-related mechanisms. We anticipate that this approach will also help to accelerate identification of candidate genes in ongoing genome-wide association studies.

## Discussion

Two of the most puzzling features of HD have been the variability in its clinical manifestation, as well as the complexity of underlying molecular changes; both are in apparent contradiction to the known monogenic nature of the disease. Light has been collectively shed on this paradox by many studies, revealing a strikingly large number of genes associated with molecular pathogenesis of HD. As it is becoming clear, progression of HD – although caused by the mutation of a single gene – is likely to be influenced by a plethora of genes with distinct functions. Moreover, studies linking genes - other than Htt - to the pathogenesis have certainly given us a better understanding of HD and have presented cues to develop novel therapies. At the same time, however, the sheer number of HD-relevant genes argues that a focus beyond studying genes as independent entities is needed and that it will be crucial to obtain a consolidated overview of the biological processes and pathways that are affected by HD.

In this study, we report the functional composition of a large set of known HD-relevant genes and thereby provide a first step towards a comprehensive view of the complex molecular landscape of HD. The basis of our work has been a curated set of 694 genes, which have been either directly or indirectly associated with HD in the HD Research Crossroads database. The composition of this set of genes was analyzed with respect to its association with molecular mechanisms. To detect processes involved in HD pathophysiology, we performed various GO and pathway analyses. Enrichment analyses enabled us to pinpoint processes overrepresented among HD-relevant genes.

Clearly, it has to be acknowledged that statistical significance is only an initial indicator for biological significance and our results need to be carefully interpreted. Statistical significance might be exaggerated by potential selection biases in HD research. Equally, processes can be pivotal for HD pathogenesis without being statistically enriched in HD-relevant genes. Also, since GO categories and KEGG pathways are not exclusive, and can have considerable overlap, significance of one category or pathway might therefore be influenced by the significance of other categories and pathways. This may lead to an overestimated significance. An example from the list of KEGG pathways illustrates this well. The pathway “Epithelial cell signaling in Helicobacter pylori infection”, which appears unrelated to HD pathogenesis, was nevertheless significantly enriched in HD-relevant genes due to inclusion of several members of the MAPK pathway. Furthermore, the results of our analysis could also be influenced by general biases underlying the examination of genes in HD research, as well as their curation in HD Crossroads. One potential bias in HD Crossroads could be a (unintended) preferential input of known drug targets, which could influence the enrichment analysis for molecular function of HD-relevant genes. This may be the case for several functions (such as kinase activity), since they also can be enriched among drug targets (see supplementary analysis in Additional file 5).

Despite these limitations, we observed that our statistical analyses are in good concordance with previous studies, which were based on the study of single or a small subset of genes. For instance, we could recover the association of protein degradation, cytotoxicity, stress response and metabolism, as well as pathways such as calcium, neurotrophin and mTOR signaling with HD. Basic molecular functions such as protein binding, RNA binding and enzyme activities (by protein kinases, peptidases, catalases and hydrolases) are also enriched. Examining their association with cellular components shows that HD-relevant proteins are predominantly located in the cytoplasm.

More interestingly, evaluation of HD-relevant genes pointed to several processes and pathways whose connections to HD have received less attention so far. For example, cell cycle and pathways such as Wnt and ErbB signaling were shown to be strongly enriched in HD-relevant genes. Our analyses suggest that these processes and pathways should be considered for further inspection and might be novel targets for therapeutic intervention for HD. Importantly, the significance of the cell cycle, as well as TLR, Wnt and ErbB pathways was not due to a single study included in the HD Crossroads. In fact, the 25 HD-relevant genes in the Wnt pathway were derived from evaluation of 20 studies. Similarly, the 18 HD-relevant genes in the ErbB pathway and the 17 genes in the TLR pathway stem from 15 and 13 distinct publications, respectively. Thus, the detected significance in enrichment can be seen as a genuine result of the integration of numerous individual studies demonstrating the value of HD Crossroads.

For our analysis, we used a filtered set of genes from HD Crossroads. We only included genes for which a causal relationship had been observed in a HD model or was indicated in an association or linkage study. However, it can be the case that genes with lower target validation scores in HD Crossroads might point to disease-relevant mechanisms of greater novelty. We have, therefore, additionally performed enrichment analyses for GO categories, KEGG pathways and Pfam families, as well as mapping to chromosomal locations for the full list of genes included in HD Crossroads. In general, the majority of enriched categories, pathways and protein families were detected as significant, irrespective of the filter for the TVS, as a comparison of the two databases has shown (Additional file 5). For the full list of genes from HD Crossroads, a larger number of significant categories, pathways and protein families were detected. This was mainly due to the fact, that they surpassed the applied threshold for a minimum number of genes (e.g., 25 for GO categories). Notably, several additional GO categories, KEGG pathways and Pfam families with apparent relevance were found. For example, the KEGG pathway “Neuroactive ligand-receptor interaction” was detected as significantly enriched, as well as the Pfam Sir 2 family, when the full HD Crossroads gene list was analyzed. Thus, the additional results might provide valuable pointers to mechanisms for further inspection and are included in the corresponding Additional files 2, 3, 4, 6, 7 and 8. It should be noted, however, that the evidence for genes with low TVS is often indirect, and thus should be treated with caution.

Remarkably, the complex nature of HD pathophysiology was highlighted by the results of our analysis. The large number of significantly enriched processes might be somewhat surprising, but it could be a direct consequence of the multi-functional role of Htt as a scaffold protein [10]. Moreover, the performed bioinformatic analyses, although extensive, have limited power to clarify causal relationships. Many detected processes might not be directly affected by mutant Htt, but may be activated as secondary responses. Accumulation of toxic mutant Htt eventually generates general stress, which leads to a wide activation of cellular response mechanisms. Due to the late disease onset and the difficulty in monitoring pre-symptomatic changes, the distinction of primary and secondary responses will remain difficult for HD, as is generally the case for late-onset neurodegenerative diseases. To distinguish cause and effect, the incorporation of time-series data will be crucial for an improved understanding of the molecular mechanisms and, finally, for dissection of the temporal order of disease progression.

In addition to the examination of the functional composition of HD-relevant genes, existing knowledge (as presented by curated causal relationships in HD Crossroads) was combined with results from linkage studies to create a list of candidates of genetic modifiers. The approach resulted in 26 HD-relevant genes located in the regions that were previously linked to age of onset. Some of these genes have high TVS, representing prime candidates for further investigation. Notably, this approach reduces the number of possible genetic modifiers by a factor of 50 and can thus greatly assist in the prioritization of targets.

## Conclusions

The results of our analyses reflect many discoveries that elucidated molecular mechanisms in HD. More remarkably, they also strongly support a functional relevance of processes, that have received little attention, or that have not been studied at all in the context of HD. We have discussed several such processes and pathways, but many others that showed statistical significance still warrant review. To this end, we have provided extensive supplementary material, including the full lists of biological processes and molecular pathways associated with HD through statistical analyses and gene annotation. Researchers are encouraged to consult the supplementary information as a resource to help define the role of different molecular processes in HD and utilize the relevant set of HD-relevant genes for further analyses. Here, it might be useful to integrate additional types of data. In our present study, we utilized gene expression to assess whether selected processes and pathways show dys-regulation in HD. We did not use expression data to assess single genes. Such an integrated approach could prove to be beneficial for further consolidation of HD-relevant genes in future studies. Similarly, the integration of chromosomal location might help to pinpoint candidate modifiers, especially if higher resolution data becomes available.

As the relevance of many genes for HD is still unknown, our analysis presents only a snapshot of current knowledge; ongoing research will give us a more complete picture. Nevertheless, our analysis can be seen as a first step towards a comprehensive list of biological processes, molecular functions and pathways associated with HD and may provide a basis for constructing more holistic disease models. Furthermore, the results presented can serve as an entry point for researchers new to HD research. We hope that our work can help to better understand the complexity of HD and contribute to the identification of potential drug targets.

## Abbreviations

BP: Biological processes; CC: Cellular compartments; FDR: False Discovery Rate; HD: Huntington's disease; GO: Gene Ontology; MF: Molecular functions; TVS: Target validation score.

## Competing interests

The authors declare that they have no competing interest.

## Authors' contributions

RK collated the set of HD-relevant genes, performed the statistical and bioinformatic analyses and prepared the original draft of the manuscript. MH performed supplementary analyses. MF conceived the study, guided the interpretation of the results and wrote the final version of the manuscript. All authors read and approved the final manuscript.

## Pre-publication history

The pre-publication history for this paper can be accessed here:

http://www.biomedcentral.com/1471-2377/12/47/prepub

## Supplementary Material

Additional file 1 **HD-relevant genes. **Excel file with the list of 694 HD-relevant genes used for analyses and their corresponding TVS and the list of genes as downloaded from HD Crossroads. Note that the first gene list also includes genes provided directly from the HD Crossroads curators, which are not included in the downloaded gene list. Click here for file

Additional file 2 **Enriched biological processes. **Excel file with three tables of biological processes and their statistical significance. A) Table with biological processes enriched in the filtered set of HD-relevant genes with FDR < 0.25. B) Table with reduced set of biological processes as defined by the GO Slim and used for Figure Figure 4. C) Table of biological processes enriched among the full set of genes curated in HD Crossroads. All tables include the GO ID of the biological process, unadjusted p-value, FDR, odds ratio, expected gene count, observed count, total number of genes associated with the biological process, as well as the gene symbols of HD-relevant genes within the GO category. To be included in table A, B or C, a minimum of 25 genes was required. Click here for file

Additional file 3 **Enriched cellular components. **Excel file with tables of cellular component categories enriched in: (A) filtered set of HD-relevant genes; and (B) the full set of genes curated in HD Crossroads. Statistical significance is given for each category. Format and filtering criteria are the same as for table S2A. Click here for file

Additional file 4 **Enriched molecular functions. **Excel file with tables of molecular function categories enriched in: (A) filtered set of HD-relevant genes; and (B) the full set of genes curated in HD Crossroads; statistical significance is given for each category. Format and filtering criteria are the same as for table S2A. Click here for file

Additional file 5 **Supplementary analyses and figures. **PDF document describing supplementary analyses and including supplementary figures S1, S2, S3, and S4. Click here for file

Additional file 6 **Enriched Pfam protein families. **Excel file with tables of Pfam protein families enriched in: (A) filtered set of HD-relevant genes; and (B) the full set of genes curated in HD Crossroads; statistical significance is given for each family. The format is the same as for table S2A, however only a minimum number of 5 genes was required. Click here for file

Additional file 7 **Enriched KEGG pathways. **Excel file with tables of KEGG pathways enriched in: (A) filtered set of HD-relevant genes; and (B) the full set of genes curated in HD Crossroads; statistical significance is given for each pathway. The format is the same as for table S2A, however only a minimum number of 10 genes was required. Click here for file

Additional file 8 **List of HD-relevant genes located in chromosomal loci, for which suggestive linkage with age of onset has been reported. **Excel file with table of HD-relevant genes located in chromosomal regions, for which suggested evidence for linkage has previously been found in two independent studies [24,26]. The table lists the TVS and chromosomal location for each gene. Click here for file
