# Supplementary material for: Huntington's Disease and its therapeutic target genes: a global functional profile based on the HD Research Crossroads database
Source: BMC Neurol. 2012 Jun 28;12:47. doi: 10.1186/1471-2377-12-47 (PMC3492045; doi:10.1186/1471-2377-12-47)
Supplement: Additional file 5 — Supplementary analyses and figures. PDF document describing supplementary analyses and including supplementary figures S1, S2, S3, and S4. [file 1471-2377-12-47-S5.pdf]

<u>Supplementary Analyses and Figures</u>

Huntington´s Disease and its therapeutic target genes: A global functional profile based on the HD Research Crossroads database

Ravi Kiran Reddy Kalathur, Miguel A. Hernández-Prieto & Matthias E. Futschik

Centro de Biomedicina Molecular e Estrutural, Campus de Gambelas, Universidade do Algarve, Faro, Portugal

1. Enrichment analyses of the unfiltered set of genes curated in HD Crossroads

For the analysis presented in the main text, a filtered set of genes from HD Crossroads was used. Filtering was performed using the Target Validation Score (TVS). Genes were only included with a corresponding TVS equal or larger than 3.0, i.e. for which a causal relationship has been deduced from experiments in a HD model, or which were indicated in an association or linkage study. In contrast, lower TVS can imply that a gene: has an altered pathway or functional activity in HD (TVS = 2.5); displays altered expression or cellular distribution in HD, or its corresponding protein binds to mutant Htt (TVS= 2.0); is active in HD-relevant brain regions or is linked to HD-associated biological mechanism (TVS=1.0); or is implicated in neurodegeneration based on genome-wide screens (TVS=0). As the inclusion of low-scoring genes may provide indication for additional disease-relevant mechanisms, enrichment analyses for GO categories, KEGG pathways and Pfam families were also carried out on the full list of genes from HD Crossroads. They include 805 genes downloaded from the HD Crossroads web-site *(http://www.hdresearchcrossroads.org)*, as well as 162 genes that were provided directly by the curators of HD Crossroads. The same thresholds for significance and minimum number of genes were applied to these analyses as for those presented in our paper. Our results can be found in the **Additional files 2-6**.

The lists of GO categories, KEGG pathways and Pfam families that were enriched were subsequently compared to the corresponding lists derived for the filtered set of genes (**Figure S1**). We found that the vast majority of categories, pathways and protein families were enriched, irrespective of the filter for the TVS. In general, a larger number of significant categories, pathways and protein families were detected for the full list of genes from HD Crossroads. A smaller number of categories, pathways or protein families were enriched only

for the filtered gene list. Inspection of the GO categories, KEGG pathways and Pfam families uniquely found in either of the two lists revealed the following: A) Most of the results unique to the unfiltered list had a number of genes close to the minimum number required, i.e., close to the threshold selected for the list, e.g. 25 genes for GO categories; B) Results unique to the filtered list generally had a FDR close the threshold of 0.25. Since their statistical significance for enrichment decreased on the unfiltered gene list, they were filtered out.

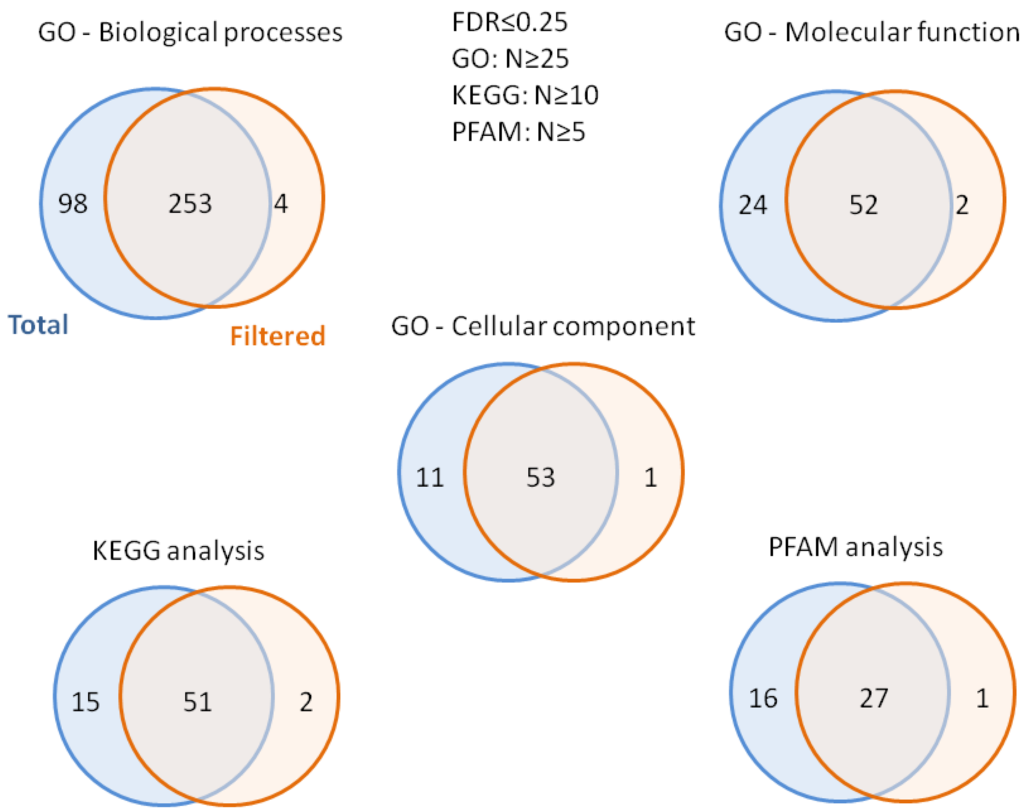

Figure S1: Comparison of enriched GO categories, KEGG pathways and Pfam protein families. Venn diagrams include the numbers of categories, pathways and protein families that were commonly or uniquely detected as enriched for the full list of genes (blue circles) and the filtered list of genes (orange circle) from HD Crossroads. Thresholds used for the different enrichment analysis are also shown.

Below we list the most significant results that are unique to the enrichment analysis for the unfiltered gene set. It appears that many genes in these categories are related to synaptic functions, but have (as yet) a low TVS, and thus, were excluded from our HD-relevant gene set.

For GO biological processes, the most significant categories that were found only for the unfiltered list were:

- regulation of synaptic transmission (N=27; FDR = 5.8 * 10^{-14})
- regulation of neurological system process (N=27; FDR = 2.4 * 10^{-13})
- neurotransmitter transport (N=29; FDR = 5.5 * 10^{-13})
- regulation of transmission of nerve impulse (N=27; FDR = 6.0 * 10^{-13})
- di-, tri-valent inorganic cation homeostasis (N=44; FDR = 4.8 * 10^{-12})

For GO cellular compartment, the most significant categories that were found only for the unfiltered list were:

- synapse (N=47; FDR = 1.9 * 10^{-10})
- plasma membrane (N=271; FDR = 1.7 * 10^{-8})
- integral to plasma membrane (N=114; FDR = 2.2 * 10^{-8})
- neuron projection (N=32; FDR = 3.5 * 10^{-8})
- intrinsic to plasma membrane (N=114; FDR = 6.6 * 10-^{8})

For GO molecular functions, the most significant categories that were found only for the unfiltered list were:

- amine binding (N=25; FDR = 4.6 * 10^{-10})
- ligand-gated ion channel activity (N=27; FDR = 7.1 * 10^{-8})
- ligand-gated channel activity (N=27; FDR = 7.1 * 10^{-8})
- receptor signaling protein activity (N=30; FDR = 3.8 * 10^{-7})
- signal transducer activity(N=182; FDR = 2.2 * 10^{-6})

For KEGG, the most significant pathways that were found only for the unfiltered list were:

- Neuroactive ligand-receptor interaction (N=80; FDR = 2.4 * 10^{-16})
- Non-small cell lung cancer (N=20; FDR = 8.2 * 10^{-6})
- Thyroid cancer (N=13; FDR = 4.7 * 10^{-5})

– Chemokine signaling pathway (N=41; FDR = 1.8 * 10^{-4})
– Prion diseases (N=13; FDR = 3.9 * 10^{-4})

For Pfam, molecular functions, the most significant unique protein families were:

– Sir2 family (N=7; FDR = 1.9* 10^{-7})
– ATP P2X receptor (N=6; FDR = 1.5 * 10^{-6})
– Sodium:neurotransmitter symporter family(N=9; FDR = 1.9 * 10^{-5})
– Nine Cysteines Domain of family 3 GPCR (N=8; FDR = 2.6 * 10^{-5})
– ATP synthase alpha/beta family, nucleotide-binding domain(N=5; FDR = 2.7 * 10^{-5})

2. Mapping of unfiltered gene set to chromosomal location with suggestive evidence for linkage with HD age of onset

In addition to the enrichment analysis, we carried out the integration with chromosomal location also for the unfiltered set of genes. Altogether, 79 genes from the unfiltered list were mapped to chromosomal locations, for which linkage with age of onset were suggested based on genome-wide scans by Li *et al*. (2003, HD MAPS study) and by Gayán *et al.* (2008). 40 genes were found in the chromosomal location indicated by the HD-MAPS study, while 77 genes were located in regions indicated by Gayán et al. In the region of overlap (2q33-35, 4p16, 5q31-32, 6q22-24), we could locate 38 genes from the unfiltered gene list (**Table S1**). **Figure S2** displays the distributions of both filtered and unfiltered genes from HD Crossroads that are located in the chromosomal regions with suggestive evidence for linkage, with respect to the two studies. The full list of genes with locations in any chromosomal region indicated by the genome-wide linkage studies can be found in the **Additional file 7.**

	EntrezID	Symbol	TVS	Chromosome	Band
**Region = 2q33-2q35**	7341	SUMO1	3	2	q33.1
	9689	BZW1	3	2	q33.1
HD-maps study: LOD =1.63	23451	SF3B1	3	2	q33.1
(at 2q33)	841	CASP8	2.5	2	q33.1
	1385	CREB1	3.5	2	q33.3
Gayan et al study: LOD =3.39	7855	FZD5	3	2	q33.3
(at 2q35)	3300	DNAJB2	3	2	q35
	8941	CDK5R2	3	2	q35
	10109	ARPC2	3	2	q35
**Region = 4p16**	6286	S100P	3	4	p16.1
	9948	WDR1	3	4	p16.1
HD-MAPS study: LOD =1.93	1816	DRD5	2	4	p16.1
	118	ADD1	3	4	p16.3
Gayan et al study: LOD =1.49	3064	HTT	4	4	p16.3
	7469	WHSC2	3	4	p16.3
	2868	GRK4	2	4	p16.3
	10815	CPLX1	2	4	p16.3
**Region = 5q31-32**	3308	HSPA4	3.5	5	q31.1
	6500	SKP1	3	5	q31.1
HD-maps study: LOD =2.02	2107	ETF1	3	5	q31.2
(at 5q31-32)	3313	HSPA9	3	5	q31.2
	7322	UBE2D2	3	5	q31.2
Gayan et al study: LOD =3.14	2676	GFRA3	2	5	q31.2
(at 5q32)	8841	HDAC3	4	5	q31.3
	10915	TCERG1	3	5	q32
	133522	PPARGC1B	3	5	q32
**Region = 6q22-24**	2444	FRK	3	6	q22.1
	6206	RPS12	3	6	q23.2
HD-maps study: LOD =2.28	9439	MED23	3	6	q23.2
(at 6q23-24)	9038	TAAR5	2	6	q23.2
	9287	TAAR2	2	6	q23.2
Gayan et al study: LOD =2.48	9288	TAAR3	2	6	q23.2
(at 6q22)	83551	TAAR8	2	6	q23.2
	134860	TAAR9	2	6	q23.2
	134864	TAAR1	2	6	q23.2
	319100	TAAR6	2	6	q23.2
	4217	MAP3K5	4	6	q23.3
	2911	GRM1	3	6	q24.3

Table S1: Candidate list of genetic modifiers. Table of genes from the unfiltered gene list that are located in chromosomal regions for which both genome-wide association studies suggest evidence of linkage with modified age of onset.

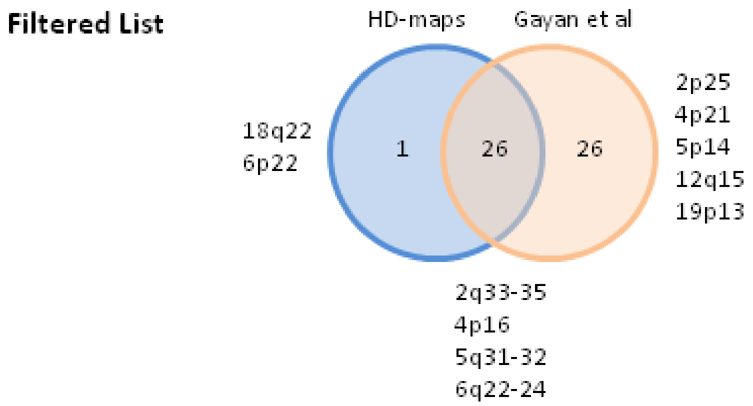

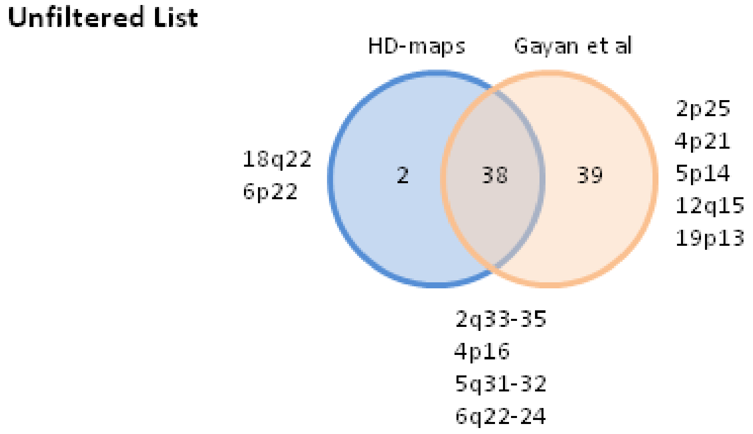

Figure S2: Number of genes in a chromosomal location with suggestive linkage to HD age of onset. The Venn diagrams depict the distribution of genes located in chromosomal regions with suggestive evidence for linkage from the two studies.

3. Assessment of potential bias of curated HD-relevant genes towards drug targets

One of the main motivations behind the implementation of HD Crossroads is to provide a platform to select gene targets, whose activity can be manipulated to treat HD. Since small molecules (”drugs”) are currently the major vesicle for the therapeutic treatment of the disease, the set of genes included in HD Crossroads might be biased towards known drug targets. This tendency might not be intentional, but may simply result from the fact that drug targeted proteins can more readily be examined for their relevance to HD. To distinguish between the available evidence for relevance and the possibility of targeting a protein by small molecules, HD Crossroads assigns a Drugability Score (DS) in addition to a Target Validation Score (TVS) to all curated genes.

To examine whether a potential bias exists in the database, we searched the list of HD-relevant genes for known drug targets. A set of 1907 known drug targets (indexed by their Entrez Gene ID) was retrieved from the DrugBank (www.drugbank.ca). In total, 197 of the HD-relevant genes were also drug targets (28%). This number is considerably higher than what we would expect by chance (N=53, assuming the total number of genes in human to be 25.000).

We also analyzed the functional composition of these known drug targets. We and found that 168 GO categories were significantly enriched in drug targets (FDR < 0.25; N ≥ 25). Subsequently, we compared this set of categories with the set of categories that we found to be enriched in HD-relevant genes. Of 54 categories enriched in HD-relevant genes, 34 were also enriched in drug targets. Examples are: *nucleotide binding* (FDR= $2.6 *10^{-25}$), *kinase activity* (FDR= $2.0 *10^{-20}$), *catalytic activity* (FDR= $6.4 *10^{-178}$), *enzyme binding* (FDR= $6.5*10^{-3}$) and *transporter activity* (FDR= $2.0 *10^{-2}$). Thus, the significance of these categories is likely to be influenced by the large number of drug targets in the set of HD–relevant genes.

For Pfam families, which were found to be enriched in HD-relevant genes, we found that only the *protein kinases* family was also enriched in drug targets (FDR= $4.9 *10^{-8}$).

Finally, we analyzed the correlation of TVS and DS, by comparing the distribution of DS for the set of genes with the same TVS (Figure S3). In general, the DS tends to increase with higher TVS. However, it should be noted that the majority of genes included in HD Crossroads have a low DS.

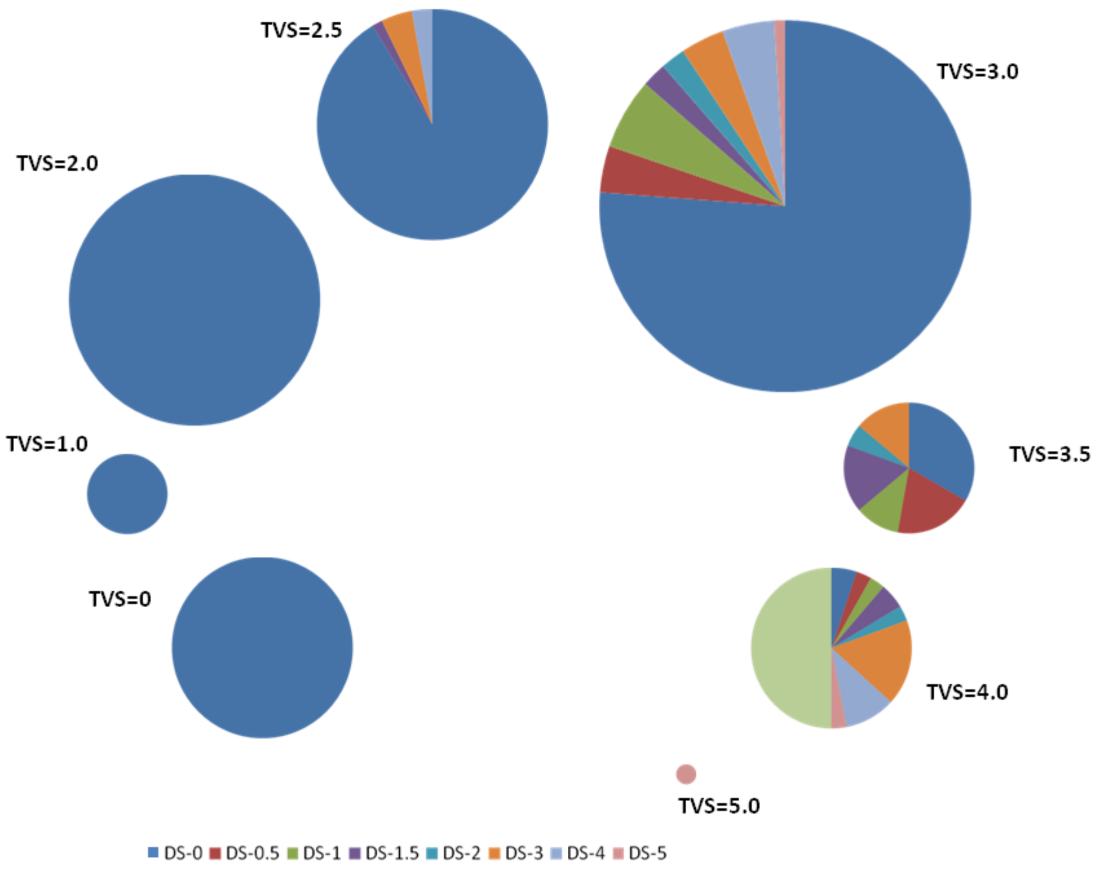

Figure S3: Distribution of Drugability scores. The pie charts depict the distribution of Drugability scores (ranging from 0.0 to 5.0) for genes with the same Target Validation scores.

A

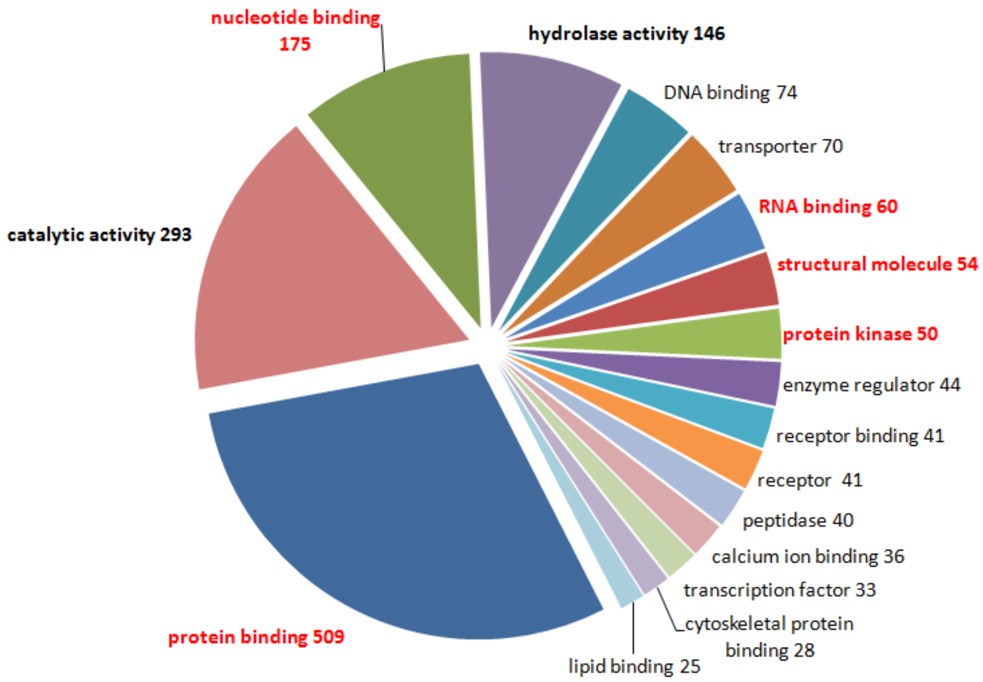

B

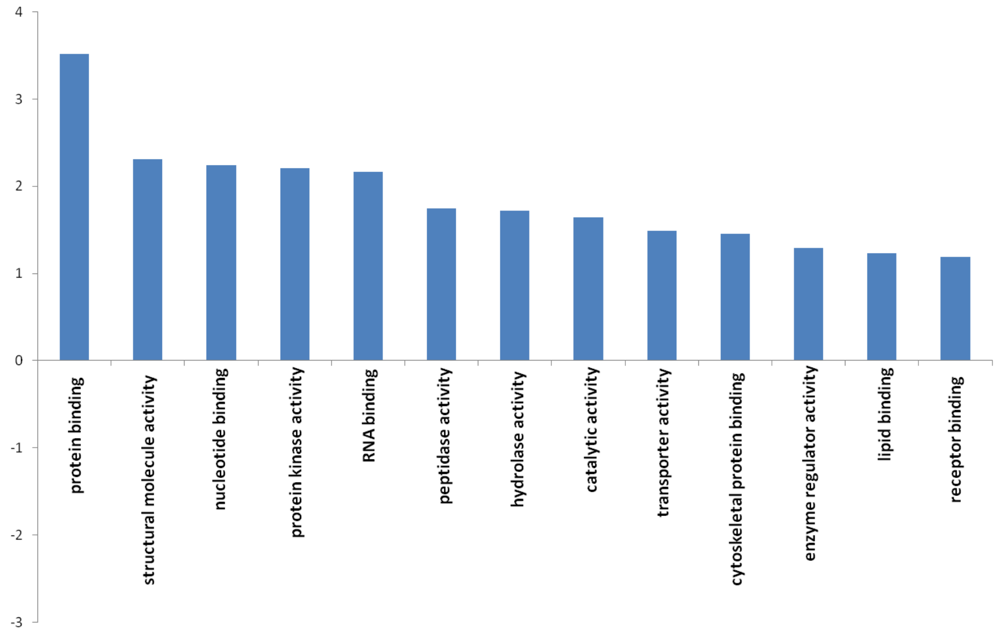

Figure S4: Distribution of HD-associated genes across molecular functions. A) The pie-chart shows the distribution of HD-associated genes linked to a reduced set of molecular processes with a minimum number of 25 genes included. GO terms for molecular processes that are significantly enriched (FDR≤0.01) are highlighted in bold. GO terms set in red indicate a corresponding odds ratio ≥ 2.0. Note that the molecular functions are not exclusive, i.e., a gene can be assigned to several functions. **B)** The bar plot displays odds ratios for enrichment by HD-associated genes in selected molecular functions.
